# Curcumin-Loaded PnBA-*b*-POEGA Nanoformulations: A Study of Drug-Polymer Interactions and Release Behavior

**DOI:** 10.3390/ijms24054621

**Published:** 2023-02-27

**Authors:** Angeliki Chroni, Thomas Mavromoustakos, Stergios Pispas

**Affiliations:** 1 Theoretical and Physical Chemistry Institute, National Hellenic Research Foundation, 48 Vassileos Constantinou Avenue, 11635 Athens, Greece; 2 Department of Chemistry, National and Kapodistrian University of Athens, Panepistimioupolis, 15771 Zografou, Greece

**Keywords:** amphiphilic block copolymers, polymeric nanocarriers, curcumin-loaded nanocarriers, drug delivery, thin film hydration method, drug encapsulation, drug release profile, drug-polymer intermolecular interactions, nuclear magnetic resonance, ultrasound

## Abstract

The current study focuses on the development of innovative and highly-stable curcumin (CUR)-based therapeutics by encapsulating CUR in biocompatible poly(n-butyl acrylate)-*block*-poly(oligo(ethylene glycol) methyl ether acrylate) (PnBA-*b*-POEGA) micelles. State-of-the-art methods were used to investigate the encapsulation of CUR in PnBA-*b*-POEGA micelles and the potential of ultrasound to enhance the release of encapsulated CUR. Dynamic light scattering (DLS), attenuated total reflection Fourier transform infrared (ATR-FTIR), and ultraviolet-visible (UV-Vis) spectroscopies confirmed the successful encapsulation of CUR within the hydrophobic domains of the copolymers, resulting in the formation of distinct and robust drug/polymer nanostructures. The exceptional stability of the CUR-loaded PnBA-*b*-POEGA nanocarriers over a period of 210 days was also demonstrated by proton nuclear magnetic resonance (^1^H-NMR) spectroscopy studies. A comprehensive 2D NMR characterization of the CUR-loaded nanocarriers authenticated the presence of CUR within the micelles, and unveiled the intricate nature of the drug-polymer intermolecular interactions. The UV-Vis results also indicated high encapsulation efficiency values for the CUR-loaded nanocarriers and revealed a significant influence of ultrasound on the release profile of CUR. The present research provides new understanding of the encapsulation and release mechanisms of CUR within biocompatible diblock copolymers and has significant implications for the advancement of safe and effective CUR-based therapeutics.

## 1. Introduction

Polymeric drug nanocarriers represent a revolutionary approach in the realm of targeted drug delivery, harnessing the intricacies of polymeric materials to encapsulate drugs at the nanoscale with unparalleled efficiency. Polymeric systems offer a multitude of possibilities for modulating the pharmacokinetics, biodistribution, and pharmacodynamics of drugs, thus opening new frontiers in the treatment of various diseases [1,2,3].

The utilization of amphiphilic block copolymers (AmBCs) in drug delivery has made notable strides in the field, with potential applications in a wide range of therapeutic areas, including cancer therapy, gene therapy, and vaccination. Their self-assembly behavior, with the ability to be functionalized in order to target specific cells, has made them a sought-after tool for drug delivery in recent years [4,5].

To date, several studies have taken advantage of poly[oligo(ethylene glycol) methyl ether methacrylate)] (POEGMA) and poly[oligo(ethylene glycol) methyl ether acrylate)] (POEGA) as hydrophilic and biocompatible polymers in the formation of micelles from AmBCs [6,7]. The incorporation of oligoethylene glycol side chains into polymers such as POEGMA and POEGA imparts biocompatibility and unique “stealth” properties that allow them to evade recognition by the immune system. These properties make POEGMA and POEGA polymers attractive candidates for use as coronas of drug carriers in various drug delivery applications [8].

The controlled delivery of hydrophobic therapeutics using AmBCs represents a cutting-edge strategy for improving the therapeutic potential of highly potent yet notoriously challenging compounds, such as curcumin (CUR) [9]. CUR boasts an array of biological activities, including antioxidant, anti-inflammatory, anticancer, and antimicrobial effects, which make it a highly promising therapeutic agent. However, its clinical application is hampered by its poor solubility, low bioavailability, and rapid metabolism in vivo, which limit its efficacy [10]. To address these limitations, researchers have turned to the development of biocompatible polymeric nanocarriers, such as micelles, to encapsulate CUR, thereby improving its solubility, stability, and bioavailability. Such polymeric nanocarriers also offer the possibility of targeted drug delivery by exploiting the enhanced permeability and retention (EPR) effect, allowing for selective delivery of CUR to specific sites in the body [11,12,13].

Several block copolymer systems, including poly(ethylene oxide)-*block*-poly(lactic acid) (PEO-*b*-PLA) [14,15], poly(ethylene oxide)-*block*-poly(caprolactone) (PEO-*b*-PCL) [16,17], poly(ethylene oxide)-poly(propylene oxide)-poly(ethylene oxide) (PEO-PPO-PEO) [18,19], etc. have been reported as effective nanocarriers for the delivery of CUR [20,21,22].

**Figure 1:**
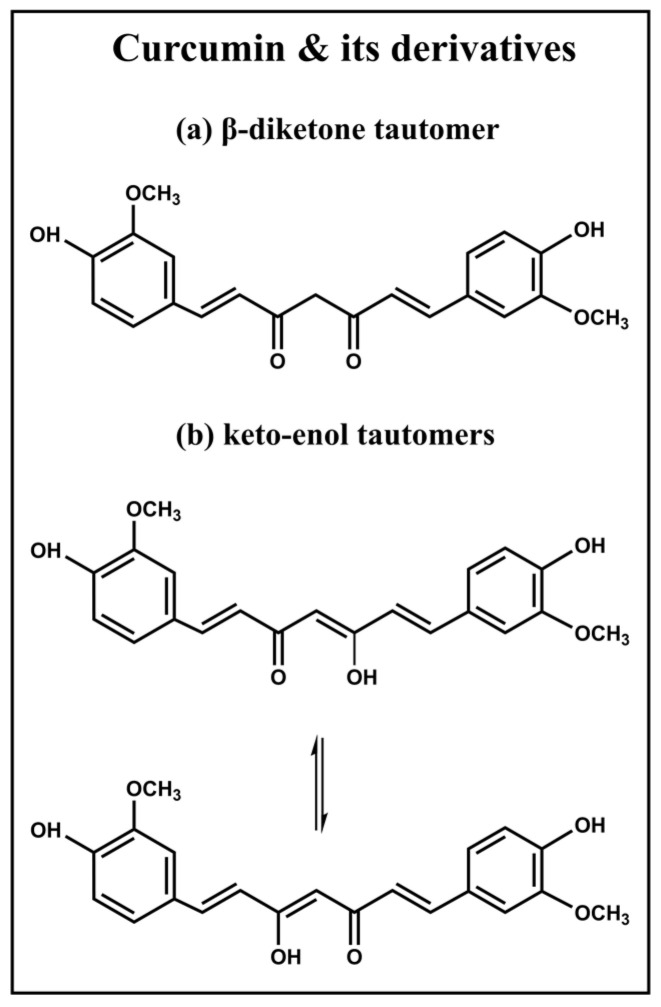
Potential solution structures of curcumin.

An in-depth comprehension of the unique chemical properties of CUR is essential to fully exploit the ability of AmBCs to deliver hydrophobic therapeutics. While its systematic nomenclature, 1,7-bis(4-hydroxy-3-methoxyphenyl)-1,6-hepatadiene-3,5-dione, might suggest that CUR exists in the form of a β diketone tautomers, (Figure 1a), studies utilizing X-ray crystal structure analysis have uncovered that, in the solid state, CUR and its bis-acetoxy derivative are present in the form of keto-enol tautomers (Figure 1b) [23]. Despite this revelation, a limited number of publications have delved into the intricacies of the keto-enol tautomers of CUR, with the majority of literature in diverse journals depicting only the β diketone tautomer [24,25,26]. In reality, there are three possible structures of CUR (Figure 1): the β-diketone tautomer, and two equivalent asymmetric keto-enol tautomers, each with its own unique characteristics, providing a fascinating subject for further study.

Other studies have shown that in solution, CUR exists mainly in the keto-enol form, particularly in solvents such as CDCl_3_ and DMSO [27,28]. The difference between the β-diketone and keto-enol tautomers of CUR lies in the hybridization of the carbon atom at a position which is either methylene or methine. Nuclear Magnetic Resonance (NMR) spectroscopy is an effective method of determining the structural differences between these forms.

NMR is a powerful and versatile technique that allows us to probe the inner workings of molecules with an unprecedented level of detail and accuracy [29]. Its ability to uncover the structural, dynamic, and interactive features of chemical species makes it an invaluable robust tool in a wide range of fields, including chemistry, biochemistry, and materials science [30]. However, the application of NMR in the analysis of drug-polymer interactions and drug encapsulation into polymers has been less explored in the literature, but it has the potential to provide valuable information. Our previous research has underscored the importance of NMR spectroscopy as a means of illustrating the relationship between the stability, encapsulation efficiency (EE%), and release kinetics of the studied systems [31,32,33]. Through the utilization of this sophisticated analytical technique, we have been able to demonstrate conclusively that the degree of interaction between the polymers and the drug plays a vital role in determining these properties. Therefore, it is reasonable to conclude that NMR spectroscopy will be increasingly used in the future studies of drug encapsulation and polymer-drug interactions, due to its ability to provide non-invasive insight into the molecular behavior of these systems.

The focus of this study is to explore the uncharted waters of CUR encapsulation into two biocompatible AmBCs with different molecular weights (M_w_) of PnBA, namely poly(n-butyl acrylate)-*block*-poly(oligo(ethylene glycol) methyl ether acrylate) (PnBA-*b*-POEGA) and to investigate the intramolecular and intermolecular interactions between the polymer and drug using 2D-NMR. At the same time, the current study aims to uncover the potential of ultrasound in facilitating the release of CUR from PnBA-*b*-POEGA micelles by performing a comparative analysis of the release profiles obtained by the dialysis method in the presence and absence of ultrasound. This research endeavors to uncover the utility of 2D-NMR in examining the encapsulation of CUR within micelles and unravel the potential of ultrasound in enhancing the release of CUR from the micelles, providing new insights for the development of innovative CUR-based therapeutics.

Thus, novel CUR-loaded PnBA-*b*-POEGA nanocarriers were developed by encapsulating CUR (20 wt. % and 50 wt. % concentration of CUR in the mixture) into PnBA_30_-*b*-POEGA_70_ and PnBA_27_-*b*-POEGA_73_ diblock copolymers (where the subscripts indicate the wt. % composition of the components). Dynamic Light Scattering (DLS) was used to characterize the size and mass of the CUR-loaded PnBA-*b*-POEGA nanocarriers. The encapsulation of CUR within the PnBA-*b*-POEGA nanoassemblies was investigated using a range of analytical techniques, including Attenuated Total Reflection Fourier Transform Infrared (ATR-FTIR) and Ultraviolet-Visible spectroscopy (UV-Vis) spectroscopies, and NMR measurements. In particular, ^1^H-NMR, correlated (2D COSY), Overhauser effect (2D NOESY), and diffusion-ordered (2D DOSY) spectroscopies were used to delve into the intermolecular interactions between the PnBA_30_-*b*-POEGA_70_ and PnBA_27_-*b*-POEGA_73_ copolymers and CUR, and to determine the self-diffusion coefficients D of the nanocarriers. Finally, the drug loading (DL%) and EE% indexes were calculated using UV-Vis spectroscopy. Subsequently, the effect of ultrasound on the release of CUR from PnBA-*b*-POEGA nanoassemblies was evaluated by using the dialysis method and comparing the release profiles obtained with and without ultrasound stimulation.

## 2. Results and Discussion

### 2.1. Synthesis of PnBA-b-POEGA Block Copolymers and Preparation of CUR-Loaded PnBA-b-POEGA Nanocarriers

**Figure 2:**
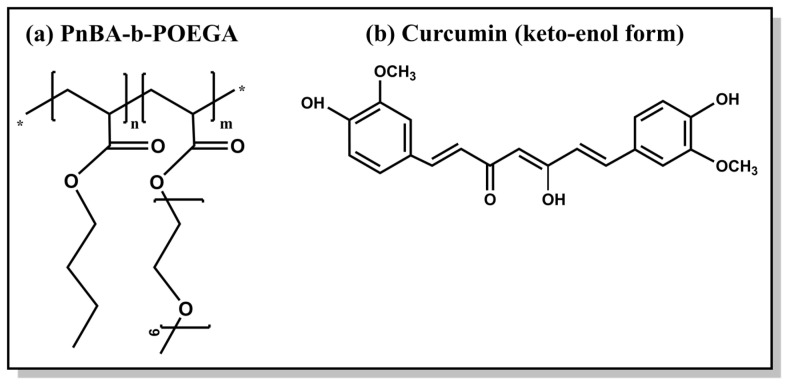
Chemical structures of (**a**) PnBA-*b*-POEGA diblock copolymers synthesized by Reversible Addition Fragmentation Chain Transfer (RAFT) polymerization and (**b**) keto-enol tautomer of CUR. The “*” refers to the repeating units of the copolymer structure.

In our previous study, we meticulously detailed the synthetic procedure and comprehensive molecular and physicochemical characterization of two distinct amphiphilic diblock copolymers, namely PnBA_30_-*b*-POEGA_70_ and PnBA_27_-*b*-POEGA_73_, which possess different M_w_ of the PnBA block (in the range of 4000 and 7800 g mol^−1^ M_w_ [34]) [31]. Table 1 shows the molecular characteristics of the PnBA-*b*-POEGA block copolymers and Figure 2 presents the chemical structures of the copolymer and CUR.

The CUR-loaded PnBA-*b*-POEGA nanocarriers were prepared using the thin film hydration method, TFHM (for further details see Materials and Methods Section 3.2). Briefly, a sufficient amount of CUR was dissolved in acetone (AC) to obtain 20 wt. % and 50 wt. % concentrations of CUR in the PnBA_30_-*b*-POEGA_70_/drug mixture, and 20 wt. % concentration of CUR in the PnBA_27_-*b*-POEGA_73_/drug mixture. It is worth noting that a 50 wt. % concentration of CUR resulted in unstable CUR-loaded PnBA_27_-*b*-POEGA_73_ nanocarriers in aqueous media over time. Finally, we filtered the drug-loaded solutions through 0.45 μm pore size filters and allowed them to stand overnight to reach equilibrium before taking any measurements.

### 2.2. Physicochemical Characterization of CUR-Loaded PnBA-b-POEGA Nanocarriers

A physicochemical evaluation of the CUR-loaded PnBA-*b*-POEGA nanocarriers and an examination of the CUR encapsulation in the polymeric PnBA micellar cores were carried out using a range of techniques including DLS, ATR-FTIR, and UV-Vis. Our previous study has reported the DLS, ATR-FTIR, and UV-Vis studies of the pure PnBA-*b*-POEGA micelles [31].

**Figure 3:**
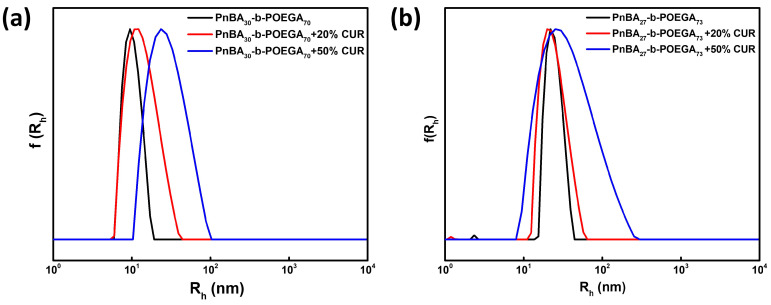
Comparative size distributions from Contin analysis at 90° for the (**a**) PnBA_30_-*b*-POEGA_70_ micelles (black line), PnBA_30_-*b*-POEGA_70_ + 20% CUR (red line) and PnBA_30_-*b*-POEGA_70_ + 50% CUR (blue line) nanocarriers and (**b**) PnBA_27_-*b*-POEGA_73_ micelles (black line), PnBA_27_-*b*-POEGA_73_ + 20% CUR (red line) and PnBA_27_-*b*-POEGA_73_ + 50% CUR (blue line) nanocarriers at 1 × 10^−3^ g ml^−1^ copolymer concentration in aqueous solutions, pH = 7, and 25 °C.

A comprehensive DLS study was carried out to determine the size distribution of CUR-loaded PnBA-*b*-POEGA nanocarriers. The DLS data presented in Figure 3 show that all solutions exhibited a symmetrical and monomodal distribution, indicating the active participation of all chains in the formation of the micellar nanostructures. Table 2 presents the values of hydrodynamic radius (R_h_), polydispersity index (PDI), and scattered light intensity of the neat PnBA-*b*-POEGA micelles and CUR-loaded PnBA-*b*-POEGA nanocarriers under the conditions of 10^−3^ g ml^−1^ concentration, pH 7, and 25 °C. It appears that upon encapsulation of CUR in PnBA_30_-*b*-POEGA_70_ and PnBA_27_-*b*-POEGA_73_ micelles, a slight broadening of the size distribution (based on the polydispersity index-PDI determined from cumulant analysis of the correlation functions obtained from the solutions studied) was observed at 20 wt. % and 50 wt. % drug concentrations. This is probably due to the increase in the hydrophobic content of the mixed/drug loaded nanocarriers.

Additionally, the DLS results indicated significant alterations in the structural features of the CUR-loaded PnBA-*b*-POEGA nanocarriers compared to the unloaded ones (Table 2). In particular, the size of the PnBA_30_-*b*-POEGA_70_ + 20% CUR and PnBA_30_-*b*-POEGA_70_ + 50% CUR nanocarriers (Figure 3a) gradually increased, indicating the efficient encapsulation of CUR into the PnBA_30_-*b*-POEGA_70_ micelles. On the other hand, the PnBA_27_-*b*-POEGA_73_ micelles loaded with 20 wt. % CUR (Figure 3b), showed a negligible decrease in size. However, when the CUR concentration was increased to 50 wt. % (Figure 3b), the size of the nanocarriers appeared to be larger than that of PnBA_27_-*b*-POEGA_73_ + 20% CUR nanocarriers, indicating successful entrapment of CUR in the polymeric micelles. Furthermore, the DLS data evidenced a significant increase in mass (based on the determined scattered intensity of the studied solutions) of the CUR-loaded PnBA-*b*-POEGA nanocarriers (Table 2), implying a successful encapsulation of the hydrophobic CUR into the PnBA-*b*-POEGA micelles. Specifically, the scattered intensity of the PnBA_27_-*b*-POEGA_73_ + 50% CUR nanocarriers increased almost five-fold compared to the bare copolymer (Table 1), indicating the presence of mixed/drug loaded nanocarriers with substantially greater mass.

Stability studies were performed using DLS to estimate the temporal stability of the resulting CUR-loaded PnBA-*b*-POEGA solutions after one week. The R_h_ and scattered intensity measurements versus time of the nanocarriers are provided in Table S1 in the Supplementary Materials. DLS studies on the mixed-drug solutions verified their stability in aqueous solutions in terms of size, PDI, and mass. In particular, the PnBA_30_-*b*-POEGA_70_ + 20% CUR, PnBA_30_-*b*-POEGA_70_ + 50% CUR, and PnBA_27_-*b*-POEGA_73_ + 20% CUR nanocarriers did not show significant variations in R_h_, PDI, and intensity, after one week, indicating stable nanostructures. Remarkably, the mixed copolymer-drug solutions of PnBA_30_-*b*-POEGA_70_ + 20% CUR, PnBA_30_-*b*-POEGA_70_ + 50% CUR, and PnBA_27_-*b*-POEGA_73_ + 20% CUR exhibited great long-term stability to the naked-eye (approximately 1 year), as no visually detectable drug precipitation was observed, under ambient room temperature conditions (around 25 °C). Unfortunately, the PnBA_27_-*b*-POEGA_73_ + 50% CUR solution was found to be unstable in aqueous media over a short period of time (approximately 2 days) and was therefore not suitable for further investigation. It is important to note that ^1^H-NMR studies were performed to evaluate the temporal stability of the CUR-loaded PnBA-*b*-POEGA solutions to complement the DLS stability assay. The methodology and results of these studies are discussed in detail in Section 2.7.

A detailed analysis of the encapsulation of CUR within the polymeric core of PnBA-*b*-POEGA micelles was undertaken using the analytical techniques of ATR-FTIR and UV-Vis spectroscopy. The ATR-FTIR and UV-Vis data are presented and discussed in Figures S1 and S2, Supplementary Materials. The ATR-FTIR spectra of the nanocarriers clearly showed new peaks specific to CUR [35,36,37]. Moreover, UV-Vis measurements further validated the successful encapsulation of CUR within the PnBA-*b*-POEGA polymeric micelles [10,38]. It can be confidently stated that the encapsulation of CUR within PnBA-*b*-POEGA micelles has been effectively demonstrated by the ATR-FTIR and UV-Vis data presented in Supplementary Materials.

### 2.3. ^1^H-NMR Studies on CUR-Loaded PnBA-b-POEGA Nanocarriers

An array of sophisticated NMR techniques was employed to unequivocally affirm the successful encapsulation of CUR within the intricately mixed drug-copolymer solutions. The ^1^H-NMR spectra for the PnBA_30_-*b*-POEGA_70_ and PnBA_27_-*b*-POEGA_73_ micelles have been reported and analyzed in our previous study [31]. Despite its vast potential, the utilization of NMR as a tool to uncover the intricacies of CUR-polymer interactions and the encapsulation of CUR within polymeric micelles remains an uncharted territory in the realm of scientific literature.

**Figure 4:**
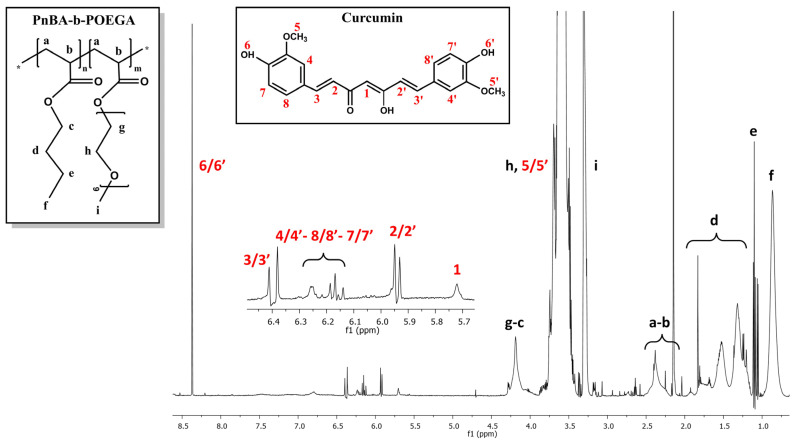
^1^H-NMR spectrum for the PnBA_30_-*b*-POEGA_70_ + 20% CUR nanocarriers in D_2_O solutions, where the black letters denote the protons of the copolymer structure (a–i), and the red numbers point to the CUR peaks.

**Figure 5:**
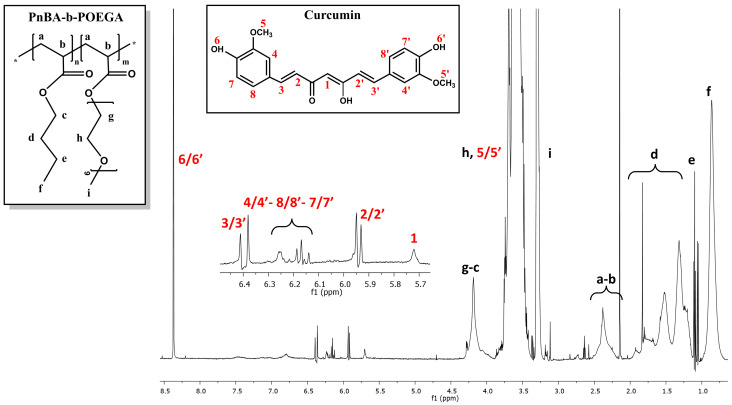
^1^H-NMR spectrum for the PnBA_30_-*b*-POEGA_70_ + 50% CUR nanocarriers in D_2_O solutions, where the black letters denote the protons of the copolymer structure (a–i), and the red numbers point to the CUR peaks.

The ^1^H-NMR spectra of the PnBA_30_-*b*-POEGA_70_ + 20% CUR and PnBA_30_-*b*-POEGA_70_ + 50% CUR nanocarriers in D_2_O solutions are observed in Figure 4 and Figure 5, while the^1^H-NMR spectrum of the PnBA_27_-*b*-POEGA_73_ + 20% CUR nanocarriers is presented in Figure S3, Supplementary Materials. The black letters in the ^1^H-NMR spectra of the nanocarriers indicate the protons of the copolymer structure, while the red numbers point to the protons of CUR. The presence of CUR in the keto-enol form in the presented drug-polymer solutions is clearly indicated by the distinct proton signal at 5.7 ppm, which is attributed to the vinylic/methine proton of the keto-enol form [39,40,41]. All the ^1^H-NMR spectra of the nanocarriers show the expected proton signals of CUR, confirming its presence in the mixed drug/copolymer solutions and thus implying the successful loading of the drug into the polymeric micelles. The chemical shifts of the proton signals of PnBA_30_-*b*-POEGA_70_ in the absence of CUR (reported in our previous work [31]) and in the presence of 20 wt. % CUR (Figure 4) and 50 wt. % CUR (Figure 5) in D_2_O solutions are summarized in Table 3. Additionally, the chemical shifts of the CUR proton signals in the presence of PnBA_30_-*b*-POEGA_70_ micelles in D_2_O solutions are summarized in Table 4. Alternative CUR-polymer mixed assemblies reported in the literature using the amphiphilic PEG-PLA copolymer exhibited different chemical shifts of CUR in CDCl_3_, whose proton signals are also presented in Table 4 [42,43,44]. In the case of the PnBA_27_-*b*-POEGA_73_ copolymer, the chemical shifts of the proton copolymer signals in the absence of CUR and in the presence of 20 wt. % CUR in D_2_O solutions are summarized in Table S2, Supplementary Materials.

### 2.4. 2D-COSY Studies on CUR-Loaded PnBA-b-POEGA Nanocarriers

**Figure 6:**
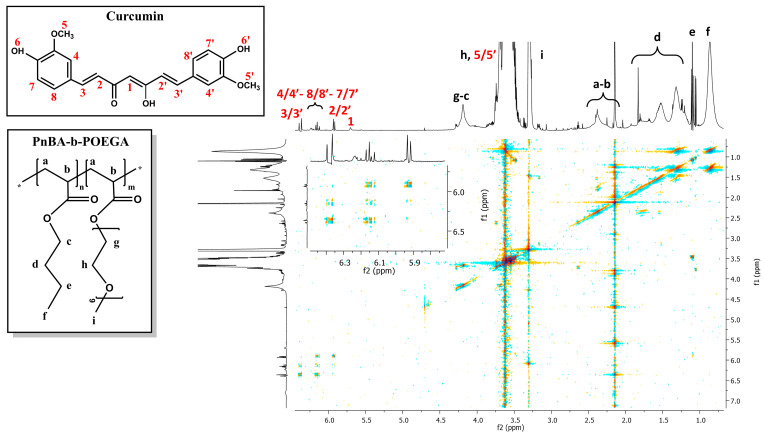
2D-COSY spectrum of the PnBA_30_-*b*-POEGA_70_ + 20% CUR nanocarriers in D_2_O solutions, where the black letters (a–i) denote the protons of the copolymer structure, and the red numbers point to the CUR peaks. The colored dots represent the cross peaks signals, which are the result of correlations between the resonances of different nuclei in the sample.

**Figure 7:**
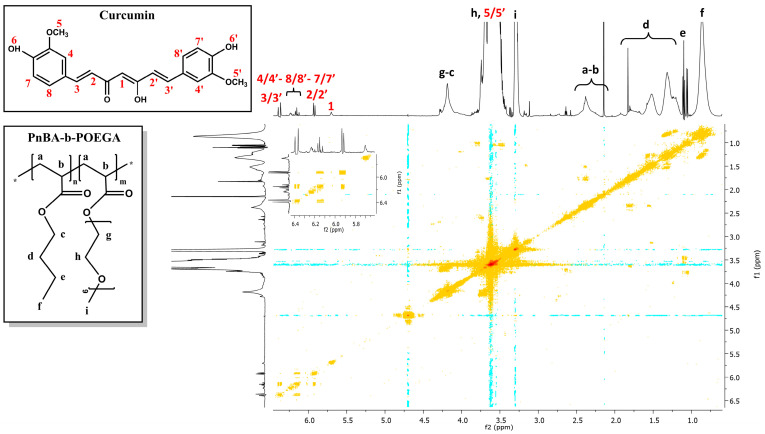
2D-COSY spectrum of the PnBA_30_-*b*-POEGA_70_ + 50% CUR nanocarriers in D_2_O solutions, where the black letters (a–i) denote the protons of the copolymer structure, and the red numbers point to the CUR peaks. The colored dots represent the cross peaks signals, which are the result of correlations between the resonances of different nuclei in the sample.

The internal structure and intramolecular interactions of PnBA-*b*-POEGA diblock copolymers with CUR moieties were analyzed using 2D-COSY measurements. The 2D-COSY spectra of the PnBA_30_-*b*-POEGA_70_ + 20% CUR and PnBA_30_-*b*-POEGA_70_ + 50% CUR nanocarriers are presented in Figure 6 and Figure 7, respectively. The 2D-COSY spectrum of the PnBA_27_-*b*-POEGA_73_ + 20% CUR nanocarriers is exhibited in Figure S4, Supplementary Materials. The figures demonstrate the protons of the copolymer structure represented by black letters, while the red numbers indicate the presence of CUR peaks. The cross peaks identified between the copolymer protons and the CUR protons in the 2D-COSY spectra indicate the presence of intramolecular interactions and provide structural characterization for both the copolymer and CUR.

### 2.5. 2D-NOESY Studies on CUR-Loaded PnBA-b-POEGA Nanocarriers

**Figure 8:**
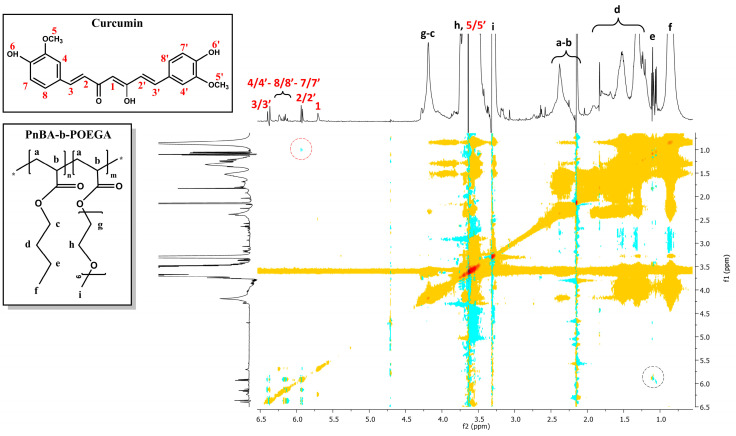
2D-NOESY spectrum of the PnBA_30_-*b*-POEGA_70_ + 20% CUR nanocarriers. The black letters (a–i) denote the protons of the copolymer structure, and the red numbers point to the CUR peaks. The colored dots represent the cross peaks signals, which are the result of correlations between the resonances of different nuclei in the sample. The colored circles represent the cross peaks between the protons of the butyl chain of PnBA at position e, and the protons of the aliphatic linker of CUR at position 2/2′.

**Figure 9:**
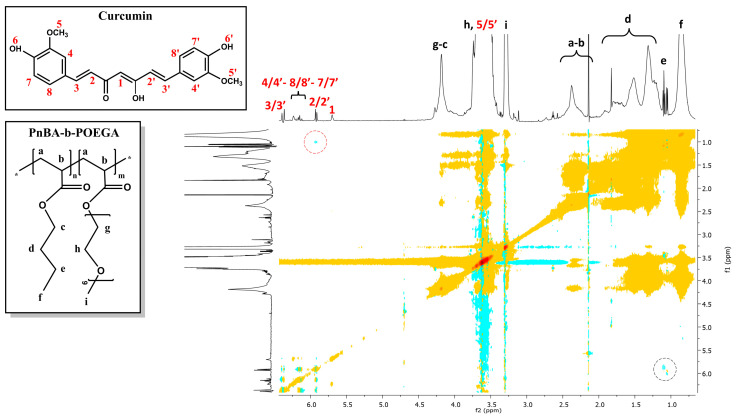
2D-NOESY spectrum of the PnBA_30_-*b*-POEGA_70_ + 50% CUR nanocarriers. The black letters (a–i) denote the protons of the copolymer structure, and the red numbers point to the CUR peaks. The colored dots represent the cross peaks signals, which are the result of correlations between the resonances of different nuclei in the sample. The colored circles represent the cross peaks between the protons of the butyl chain of PnBA at position e, and the protons of the aliphatic linker of CUR at position 2/2′.

The relationship between the copolymer and CUR was further investigated using 2D-NOESY experiments to confirm the successful incorporation of CUR into the PnBA-*b*-POEGA micelles and to examine the proximity of the copolymers and the drug. 2D-NOESY results of the PnBA_30_-*b*-POEGA_70_ + 20% CUR and PnBA_30_-*b*-POEGA_70_ + 50% CUR nanocarriers are depicted in Figure 8 and Figure 9, whereas the 2D-NOESY spectrum of the PnBA_27_-*b*-POEGA_73_ + 20% CUR nanocarriers is exhibited in Figure S5, Supplementary Materials. All data show a weak binding between CUR and the PnBA hydrophobic core. Particularly, only the aliphatic linker of CUR (2/2′) is in close proximity to the butyl chain of PnBA, at position e, indicating that the hydrophobic interactions are exerted by the carbon aliphatic linker of CUR and the butyl chain of PnBA.

The 2D-NOESY results indicate that the interactions between CUR and the polymeric micelles are weak and non-intense, which could be related to a low encapsulation strength. This suggests that CUR is not optimally encapsulated within the micelles. It is possible that a significant proportion of the encapsulated drug may be situated on the core-corona interface of the micelles, rather than within the hydrophobic core, leading to a lack of proximity and interaction with the polymer molecules, or CUR molecules are not well-dispersed within the polymer matrix. It is therefore essential to investigate the encapsulation efficiency of CUR within the micelles, in order to fully understand the drug-polymer interactions and optimize the drug delivery. However, the encapsulation strength of a drug within a polymer matrix can also play a decisive role in determining the release kinetics of said drug. For a more detailed discussion of this concept, please refer to Section 2.8.

It is noteworthy to mention that our prior research, using the same polymer but a more hydrophilic drug, namely losartan potassium, yielded eminent correlations between PnBA-*b*-POEGA and LSR in D_2_O, indicating a robust association between the polymer and drug. The discrepancy may be attributed to the less hydrophobic nature of losartan (compared to the highly hydrophobic CUR), which enhances its encapsulation into the relatively hydrophobic and pliable PnBA block (considering its low T_g_ value) [31].

### 2.6. 2D-DOSY Studies on CUR-Loaded PnBA-b-POEGA Nanocarriers

**Figure 10:**
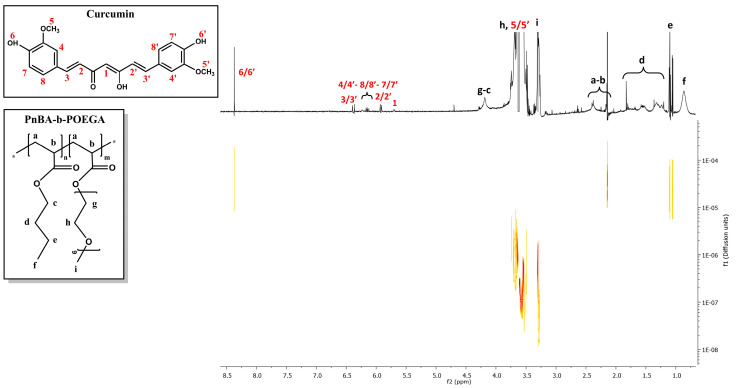
2D-DOSY spectrum of PnBA_30_-*b*-POEGA_70_ + 20% CUR nanocarriers. The black letters (a–i) denote the protons of the copolymer structure, and the red numbers point to the CUR peaks.

**Figure 11:**
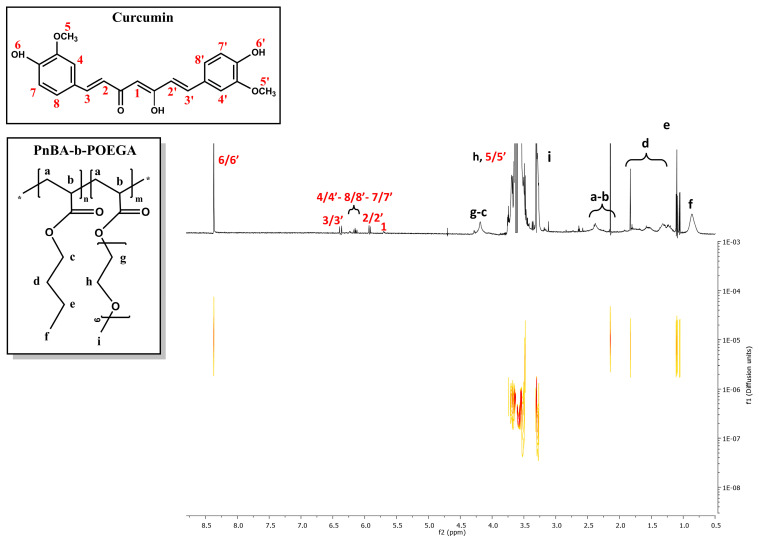
2D-DOSY spectrum of PnBA30-b-POEGA70 + 50% CUR nanocarriers. The black letters (a–i) denote the protons of the copolymer structure, and the red numbers point to the CUR peaks.

2D-DOSY experiments were also performed to determine the self-diffusion coefficients D of the CUR-loaded nanocarriers and to identify drug association with micelles and the presence of assemblies. Figure 10 and Figure 11 indicate the diffusion experiments for the PnBA_30_-*b*-POEGA_70_ + 20% CUR and PnBA_30_-*b*-POEGA_70_ + 50% CUR nanocarriers. The 2D-DOSY spectrum of the PnBA_27_-*b*-POEGA_73_ + 20% CUR nanocarriers is presented in Figure S6, Supplementary Materials. Particularly, three series of traces with intermediate diffusion coefficients (D = 3.31 × 10^−7^ m^2^ s^−1^ and D = 3.5 × 10^−7^ m^2^ s^−1^) appear in Figure 10, which can be attributed to the diffusion of block copolymer micelles and unimolecular block copolymers, respectively. The trace with the highest coefficient (D = 3.7 × 10^−5^ m^2^ s^−1^), can be referred to the diffusion of CUR. The significant disparity in the diffusion coefficient between the free form of CUR and its micelle-bound counterpart implies a robust exchange rate between the two states. The high exchange rate is indicative of the dynamic nature of the drug-polymer interactions, and it could provide a valuable insight into the optimization of drug delivery systems and the underlying mechanisms of drug-polymer interactions.

Regarding Figure 11, a triple series of traces with medium constants (D = 4.0 × 10^−7^ m^2^ s^−1^ and D = 6.38 × 10^−6^ m^2^ s^−1^) are evident and may be related to the diffusions of block copolymer micelles and monomeric units, respectively. It is likely that the trace with the highest constant (D = 1.04 × 10^−5^ m^2^ s^−1^) indicates a relatively high exchange rate between the free and micelle-bound state of CUR.

### 2.7. ^1^H-NMR Stability Studies on CUR-Loaded PnBA-b-POEGA Nanocarriers

^1^H-NMR studies were repeated to estimate the temporal stability of the mixed-drug solutions, in D_2_O solutions, over a period of 210 days (7 months). Specifically, ^1^H-NMR stability studies were carried out on the nanocarriers suspended in D_2_O solutions, with the aim of detecting possible changes to their initial ^1^H-NMR spectra (exhibited in Section 2.3), over time. By analyzing the variations in integrated intensity, half-width, and chemical shift of the proton signals compared to the original ones, we gained valuable insights into the structural integrity of the nanocarriers—a crucial aspect for their use in drug delivery.

**Figure 12:**
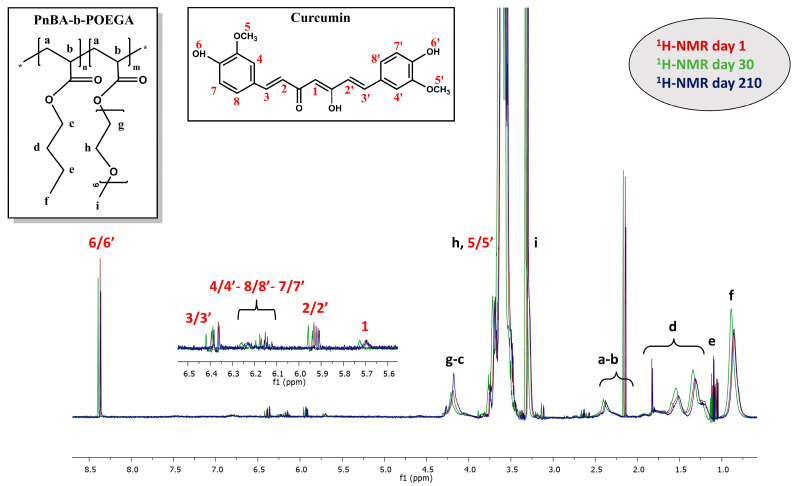
^1^H-NMR stability studies for the PnBA_30_-*b*-POEGA_70_ + 50% CUR nanocarriers in D_2_O solutions, on days 1 (red line), 30 (green line), and 210 (blue line), where the black letters denote the protons of the copolymer structure (a–i), and the red numbers point to the CUR peaks.

The ^1^H-NMR studies of the PnBA_30_-*b*-POEGA_70_ + 20% CUR, PnBA_30_-*b*-POEGA_70_ + 50% CUR, and PnBA_27_-*b*-POEGA_73_ + 20% CUR nanocarriers have conclusively proven the stability of these solutions in aqueous environments over an extended period of 210 days. The characteristic peaks of CUR were consistently observed throughout the duration of the study, attesting to the chemical integrity and structural stability of the drug in question. Indicatively, a comparison of the ^1^H-NMR spectra for the PnBA_30_-*b*-POEGA_70_ + 50% CUR nanocarriers in D_2_O solutions on days 1, 30, and 210 is illustrated in Figure 12.

Upon initial observation, the comparative ^1^H-NMR spectra of the PnBA_30_-*b*-POEGA_70_ + 50% CUR nanocarriers (Figure 12) clearly showed all the characteristic peaks of CUR over a period of 210 days, indicating stable nanostructures over time. Notably, a slight decrease in the integrated intensity of the CUR proton signals was observed at day 210 compared to day 1, possibly indicating a small decrease in the solubility of the drug as a result of some gradual release from the micelles. Overall, the results of the ^1^H-NMR studies provided a significant understanding of the stability of these nanocarriers over an extended period of time, making them suitable for further development in the field of drug delivery.

### 2.8. Encapsulation and Release Studies

Ultrasound-mediated release experiments with polymeric nanocarriers offer a non-invasive, safe, and cost-effective means of controlled drug delivery, while minimizing the undesirable side effects associated with traditional therapies [45,46,47]. Ultrasound has the ability to penetrate a wide range of tissues and induce both mechanical and thermal effects. It can also be used to enhance the release of CUR from micelles by creating cavitation bubbles that can disrupt the micelle structure and increase membrane permeability [48].

In light of these benefits, an investigation of the impact of ultrasound on the release of CUR from PnBA-*b*-POEGA nanoassemblies was conducted, by utilizing the dialysis method and comparing the release profiles obtained, in the presence and absence of ultrasound. The highly stable PnBA_30_-*b*-POEGA_70_ + 50% CUR and PnBA_27_-*b*-POEGA_73_ + 20% CUR nanocarriers were hand-picked for the study of release kinetics and revealed a dynamic and intriguing release profile of CUR, depending on the presence or absence of ultrasound. DL% and EE% were calculated by UV-Vis spectroscopy using Equations (1) and (2), and are summarized in Table 5. The increased hydrophobicity of CUR seems to favor the encapsulation of CUR in the hydrophobic, relatively fluid PnBA core, resulting in high EE% values for both PnBA_30_-*b*-POEGA_70_ + 50% CUR (88%) and PnBA_27_-*b*-POEGA_73_ + 20% CUR (86%) nanocarriers.

The assessment of drug delivery systems requires a comprehensive understanding of both encapsulation efficiency and interaction data in order to optimize drug delivery and achieve the desired therapeutic outcomes. Specifically, in cases where the nanocarriers present high encapsulation efficiency values, but display weak NOESY signals, as occurring in the current study, it is imperative to consider both sets of data in tandem. This discrepancy can be attributed to the fact that the drug is well-encapsulated within the polymeric micelles, but the intermolecular interactions between the drug and the polymer are weak, resulting in weak NOESY signals and low encapsulation strength. However, the encapsulation strength of a drug within a polymer matrix can play a crucial role in determining the release kinetics of a drug.

**Figure 13:**
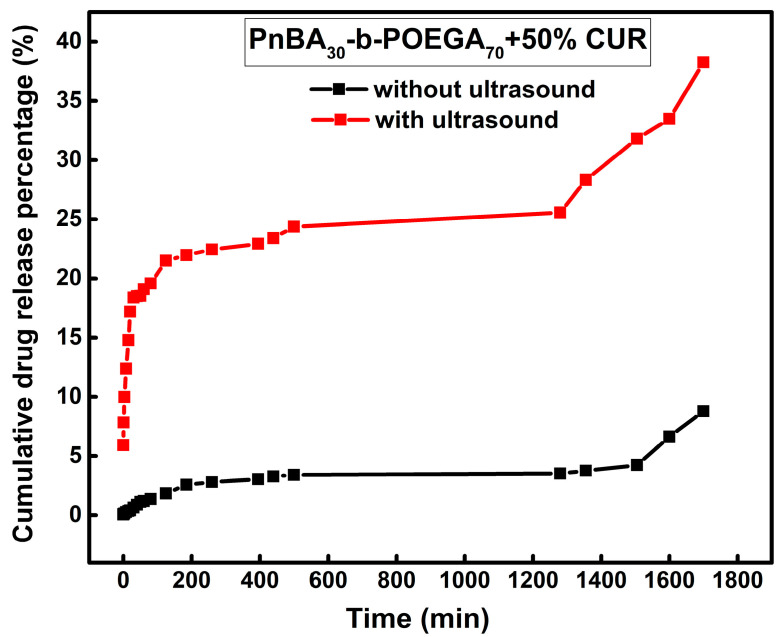
Release of 50 wt. % CUR from PnBA_30_-*b*-POEGA_70_ micelles in aqueous media with (red line) and without (black line) the application of ultrasound.

**Figure 14:**
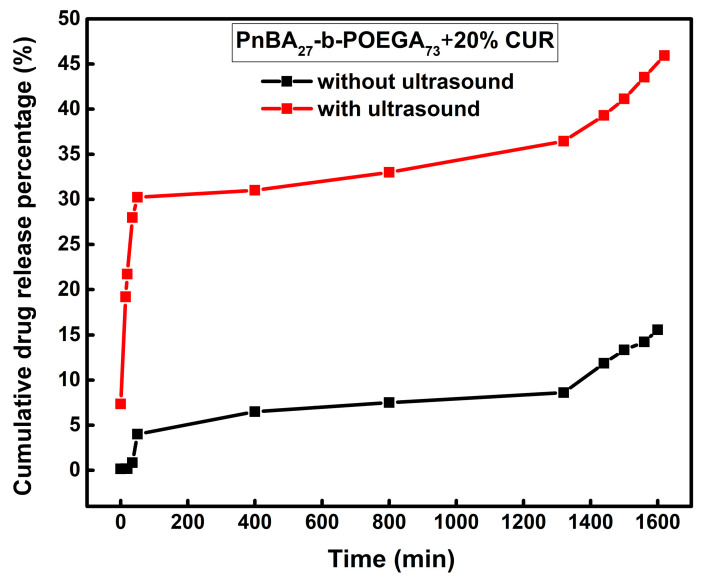
Release of 20 wt. % CUR from PnBA_27_-*b*-POEGA_73_ micelles in aqueous media with (red line) and without (black line) the application of ultrasound.

The cumulative drug release percentage versus time for the PnBA_30_-*b*-POEGA_70_ + 50% CUR and PnBA_27_-*b*-POEGA_73_ + 20% CUR nanocarriers without (a) and with (b) ultrasound are provided in Figure 13 and Figure 14. Without ultrasound (Figure 13, black line), the release profile started with a minuscule and steady increase in released CUR (1%) in the first hour, followed by a slower release (3%) up to 8 h, and then transitioned to a plateau phase, before culminating in a relatively brisk and sustained release of CUR (9%) throughout the duration of the measurement. However, when ultrasound was applied, the release profile underwent a drastic transformation. An initial surge in CUR release (18%) was observed within the first 30 min (Figure 13, red line), followed by a relatively gradual and slow increase in released CUR until reaching a plateau, and then, a continuous and rapid release of CUR (38%) within 30 h.

Additionally, the PnBA_27_-*b*-POEGA_73_ + 20% CUR nanocarriers displayed a distinct release profile in the absence of ultrasound (Figure 14, black line), featuring two stages. An initial and steady increase in released CUR was observed during the first 35 min, reaching 4%, followed by a slow and continuous release of CUR (15%), without reaching a plateau, until the end of the experiment. On the other hand, ultrasound stimulation (Figure 14, red line) induced an initial burst release of CUR (30%) during the first 50 min, followed by a slow and gradual release of CUR at longer times, and then a faster and continuous release of CUR (46%) until the end of the measurement.

In summary, the relatively low release of CUR suggests that a considerable portion of the encapsulated drug is firmly bound to the hydrophobic cores of the polymeric micelles and is challenging to be released. However, the observed initial burst release may indicate that the drug molecules are weakly held within the matrix and that a fraction of the encapsulated drug is located at the core/corona interface of the micelles and is thus more accessible and prone to release. In addition, given the high encapsulation efficiency of the nanocarriers, the weak drug-polymer interactions, and their low encapsulation strength, it is possible that the CUR molecules are not well-dispersed within the polymer matrix, leading to a faster drug release. These observations emphasize the complexity of drug encapsulation and release within polymeric micelles and the importance of understanding the underlying mechanisms to optimize drug delivery.

**Figure 15:**
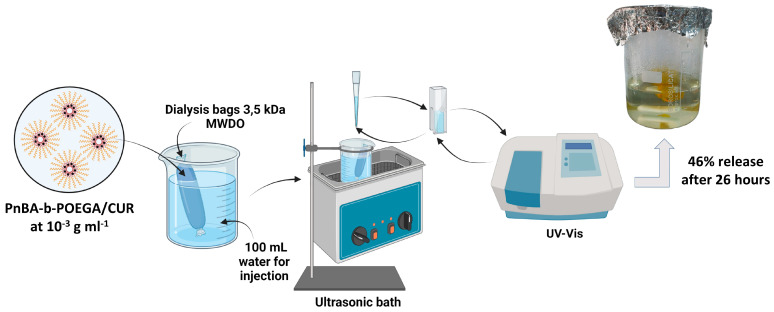
Schematic illustration of the ultrasound release studies of the PnBA_27_-*b*-POEGA_73_ + 20% CUR nanocarriers, which demonstrated a maximum release rate of 46% CUR in aqueous solutions after 26 h.

Overall, the presented release results demonstrate the significant influence of ultrasound on the release profile of CUR from PnBA_30_-*b*-POEGA_70_ and PnBA_27_-*b*-POEGA_73_ micelles. A schematic illustration of the ultrasound release studies of the PnBA_27_-*b*-POEGA_73_ + 20% CUR nanocarriers, which showed a maximum release rate of 46% CUR in aqueous solutions after 26 h, is shown in Figure 15.

## 3. Materials and Methods

### 3.1. Materials

The following materials were used in this study: curcumin (CUR, Merck, Athens, Greece), acetone (AC, 99.5%, Sigma-Aldrich, Athens, Greece), water for injection (WFI, 99%, Sigma-Aldrich, Athens, Greece), and deuterium oxide (D_2_O, 99%, Sigma-Aldrich, Athens, Greece). The synthesis of PnBA-*b*-POEGA block copolymers was performed by reversible addition fragmentation chain transfer (RAFT) polymerization using 2,2-azobis (isobutyronitrile) (AIBN, 98% Sigma-Aldrich, Athens, Greece) as the initiator, PnBA homopolymers (synthesized in 4000 and 7800 g mol^−1^ molecular weight [34]) as macro-chain transfer agents for the polymerization of the OEGA monomer (Μ_w_ = 480 g/mol, Sigma-Aldrich Athens, Greece), and 1,4-dioxane as the solvent [31].

### 3.2. Preparation of CUR-Loaded PnBA-b-POEGA Nanocarriers

**Figure 16:**
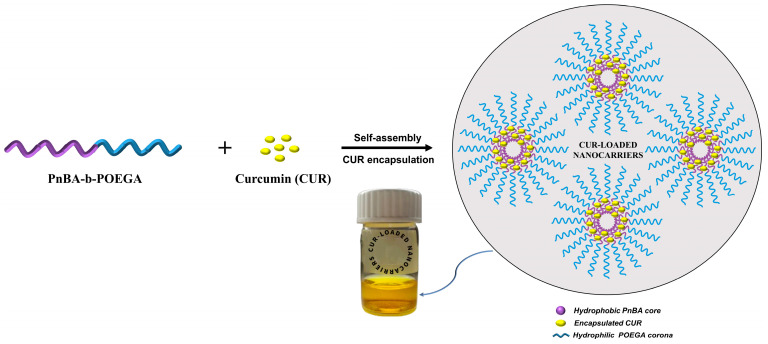
Schematic illustration for the preparation of CUR-loaded PnBA-*b*-POEGA nanocarriers using the TFHM in aqueous solutions.

In our previous work, the synthesis of PnBA-*b*-POEGA block copolymers by RAFT polymerization and their self-assembly behavior in aqueous solutions have been described in detail [31]. The CUR-loaded PnBA-*b*-POEGA nanocarriers were prepared using the TFHM [49]. The concentrations of CUR used in the copolymer/drug mixture are based on theoretical drug concentrations routinely used for polymeric amphiphiles [50,51]. Particularly, an appropriate amount of CUR was dissolved in AC to prepare 20 wt. % and 50 wt. % concentration of CUR in the PnBA_30_-*b*-POEGA_70_/drug mixture and 20 wt. % concentration of CUR in the PnBA_27_-*b*-POEGA_73_/drug mixture (50 wt. % concentration of CUR produced CUR-loaded PnBA_27_-*b*-POEGA_73_ nanocarriers with poor stability in aqueous media over time). The combination of copolymer and CUR stock solution was prepared by mixing the suitable quantities, and then transferred into a flask. The flask was then placed in a rotary evaporator, where AC was efficiently evaporated to form a thin film of copolymer and CUR on the inner walls of the flask. The appropriate amount of filtered WFI was then added to the flask and stirred until the thin film was completely dissolved. The concentrations of the aqueous stock solutions were 10^−3^ g ml^−1^. Figure 16 illustrates the preparation of CUR-loaded PnBA-*b*-POEGA nanocarriers using the TFHM method, in aqueous solutions.

### 3.3. Drug Loading and Encapsulation Efficiency Calculations of CUR

The percentage of CUR encapsulated in PnBA-*b*-POEGA micelles was estimated by UV-Vis (Perkin-Elmer, Lambda 19 spectrophotometer, Waltham, MA, USA) spectroscopy. The concentration of CUR was determined utilizing a CUR calibration curve in AC. DL% is defined as the ratio of the total mass of drug present in a micelle to the total mass of the micelle. It is typically expressed as a percentage and is calculated by dividing the total mass of drug entrapped in the micelle by the total mass of the micelle. EE% is defined as the ratio of the total mass of drug effectively entrapped in a micelle to the total mass of drug added to the micelle. It is also typically expressed as a percentage and is calculated by dividing the total mass of encapsulated drug by the total mass of drug added to the micelle.

The values of DL% and EE% are provided in Table 5, and have been calculated using the following equations:

DL% = [encapsulated drug/total micelles weight] × 100(1)

EE% = [encapsulated drug/total drug added] × 100(2)

### 3.4. Release Studies

The limited solubility of CUR in water, which is only 4.2 μg mL^−1^, is an important factor to consider in studying the release of CUR from PnBA-*b*-POEGA micelles, as its solubility affects the availability of the drug for release [52,53]. Therefore, the release of CUR from PnBA-*b*-POEGA micelles was studied using the dialysis bag method (simulating physiological conditions in the body) in WFI, both in the presence and absence of ultrasound. For the release assay, 1.5 mg CUR was used to prepare the PnBA_30_-*b*-POEGA_70_ + 50% CUR nanocarriers, while 0.27 mg CUR was added to the PnBA_27_-*b*-POEGA_73_ + 20% CUR mixed solution. Specifically, 5 mL of each sample was placed in dialysis bags of 3.5 kDa MWDO. The dialysis bags were then inserted into 100 mL of filtered WFI and placed in a SOLTEC, SONICA 3300ETH-S3 ultrasonic bath sonicator (in the case of ultrasound) or a magnetic stirring plate. Samples were taken from the external solution at specified time intervals, and the aqueous solution was replenished to its initial volume each time to maintain constant conditions. The amount of CUR released at different time intervals, up to 30 h, was determined at λ_max_ = 420 nm based on a CUR calibration curve in AC, using UV-Vis spectroscopy (Perkin-Elmer, Lambda 19 spectrophotometer, USA).

### 3.5. NMR Sample Preparation

The CUR-loaded PnBA-*b*-POEGA nanocarriers were prepared by the TFHM method in D_2_O solutions as follows: 1 mg of each PnBA-*b*-POEGA copolymer was dissolved in 1 mL of D_2_O. The distribution of the drug within the PnBA-*b*-POEGA micelles was investigated by introducing an appropriate amount of CUR into the final copolymer-drug mixture.

### 3.6. Evaluation

^1^H-NMR spectroscopic analysis of CUR-loaded PnBA-*b*-POEGA nanocarriers was performed using a 600 MHz NMR spectrometer (Agilent Technologies, Palo Alto, CA, USA), controlled by Vjnmr rev. 3.2A software, and a 5 mm HCN cold probe. Tetramethylsilane (TMS) was used as the internal standard and D_2_O as the solvent. Spectra were recorded with 65,536 points, 90° pulse angle, 10 s relaxation delay, and 32 repetitions. Correlated spectroscopy (2D-COSY), Overhauser (2D NOESY), and diffusion-ordered (2D DOSY) experiments were also performed on the 600 MHz NMR spectrometer. The NOESY spectra were recorded at different mixing times with 4096 × 200 points, 1 s relaxation delay, and 32 repetitions per spectrum. The DgcsteSL cc sequence was used to record DOSY spectra with 65,536 points, 1 s relaxation delay, and 16 repetitions. Twenty-four gradient strengths between zero and 60 gauss/cm were used. All spectra were recorded at 25 °C and chemical shifts were referenced with respect to the lock frequency and reported relative to TMS.

The DLS measurements were conducted using the ALV/CGS-3 Compact Goniometer System from ALV GmbH, Langen (Hessen, Germany), which includes a 288-channel ALV-5000/EPP multi-tau digital correlator, an ALV/LSE-5003 light scattering electronics unit for stepper motor drive, and limit switch control. The light source is a JDS Uni-phase 22-mW He-Ne laser with a wavelength of 632.8 nm. The size data and figures presented in the manuscript were taken as the average of five measurements per concentration/angle at 90 degrees. Correlation functions were analyzed using the cumulants method and CONTIN software (ALV-Correlator Software Version 3.0.) from ALVGmbH, Hessen, Germany. All solutions were filtered through 0.45 μm hydrophilic PTFE filters (Millex-LCR from Millipore, Billerica, MA, USA) prior to measurements.

UV−Vis absorption spectra of free CUR, CUR-loaded PnBA-*b*-POEGA nanocarriers, and aqueous solutions of the released CUR were recorded between 200 and 600 nm using a Perkin Elmer (Lambda 19) UV–Vis–NIR spectrophotometer (Waltham, MA, USA). The CUR-loaded PnBA-*b*-POEGA nanocarriers were diluted to obtain an absorbance value of less than 1. It is worth noting that the absorption at 260 nm and 422 nm indicates the presence of CUR and not of the copolymer, as confirmed by measurements on the neat copolymers.

Mid-infrared (IR) spectra of CUR-loaded PnBA-*b*-POEGA nanocarriers were recorded in the region 550–4000 cm^−1^ on an Equinox 55 FTIR spectrometer (Bruker, Billerica, MA, USA), which was equipped with a single reflection diamond ATR accessory from SensIR Technologies (Du-raSamp1IR II).

## 4. Conclusions

Novel and highly stable CUR-loaded nanocarriers were successfully developed by encapsulating CUR in two biocompatible AmBCs, PnBA_30_-*b*-POEGA_70_ and PnBA_27_-*b*-POEGA_73_, and thoroughly investigated using a variety of techniques. The utilization of advanced analytical methods, such as DLS, ATR-FTIR, and UV-Vis, allowed us to confirm the successful encapsulation of CUR within the inner hydrophobic segments of the copolymers, resulting in the formation of durable drug/polymer nanostructures.

Moreover, ^1^H-NMR stability studies of the CUR-loaded PnBA-*b*-POEGA nanocarriers revealed their exceptional stability over a period of 210 days, lending further credence to the chemical integrity and structural stability of CUR. A comprehensive 2D NMR characterization of the CUR-loaded nanocarriers further authenticated the presence of CUR within the micelles and unveiled the intricate nature of the drug-polymer interactions.

UV-Vis results also indicated high encapsulation efficiency values for the CUR-loaded nanocarriers and revealed a significant influence of ultrasound on the release profile of CUR. Specifically, release studies of PnBA_27_-*b*-POEGA_73_ + 20% CUR nanocarriers showed a maximum release rate of 46% CUR in the presence of ultrasound and 16% CUR in the absence of ultrasound, within 26 h.

In conclusion, this study has provided a comprehensive understanding of the encapsulation and release mechanisms of CUR within biocompatible diblock copolymers, through the use of advanced analytical techniques and ultrasound. These findings have significant implications for the advancement of safe and efficacious drug delivery systems in the biomedical field and open the door to new opportunities in the field of nanomedicine.

## Figures and Tables

**Table 1:** Molecular characteristics of the PnBA-*b*-POEGA block copolymers.

Sample	M_w_ ^a^ (× 10^4^ g mol^−1^)	wt. % PnBA ^b^
PnBA_30_-*b*-POEGA_70_	1.29	30
PnBA_27_-*b*-POEGA_73_	2.86	27

^a^ Determined by Size Exclusion Chromatography (SEC) in tetrahydrofuran (THF)/3%v Triethylamine (Et_3_N), ^b^ Determined by ^1^H-NMR in deuterated chloroform (CDCl_3_).

**Table 2:** Results for the PnBA-*b*-POEGA micelles and CUR-loaded PnBA-*b*-POEGA nanocarriers in aqueous solutions.

Sample	R_h_ ^a^ (nm)	PDI ^a^	Intensity ^a^ (a.u)
PnBA_30_-*b*-POEGA_70_	10	0.12	167
PnBA_30_-*b*-POEGA_70_ + 20% CUR	14	0.21	210
PnBA_30_-*b*-POEGA_70_ + 50% CUR	28	0.25	470
PnBA_27_-*b*-POEGA_73_	24	0.15	960
PnBA_27_-*b*-POEGA_73_ + 20% CUR	22	0.16	926
PnBA_27_-*b*-POEGA_73_ + 50% CUR	35	0.31	4000

^a^ Determined by DLS at measuring angle 90°.

**Table 3:** ^1^H-NMR chemical shifts of PnBA_30_-*b*-POEGA_70_ copolymer: (**a**) in the absence of CUR and in the presence of (**b**) 20 wt. % CUR and (**c**) 50 wt. % CUR in D_2_O solutions.

	^1^H-NMR Chemical Shifts of PnBA_30_-*b*-POEGA_70_ Copolymer (ppm)		
Protons of Polymer Structure	(a) **In the Absence of CUR**	(b) **In the Presence of 20 wt. % CUR**	(c) **In the Presence of 50 wt. % CUR**
Ha-b	2.13	2.14	2.14
Hc	3.94	4.05	4.05
Hd	1.51	1.52	1.52
He	1.1–1.34	1.1–1.31	1.09–1.32
Hf	0.86	0.86	0.86
Hg	4.17	4.19	4.18
Hh	3.60	3.62	3.62
Hi	3.28	3.31	3.30

**Table 4:** ^1^H-NMR chemical shifts of CUR: in the presence of (**a**) PnBA_30_-*b*-POEGA_70_ in D_2_O and in the presence of (**b**) PEG-PLA in CDCl_3_.

	^1^H-NMR Chemical Shifts (ppm)	
Protons of CUR Structure	(a) **In the Presence of PnBA_30_-*b*-POEGA_70_**	(b) **In the Presence of PEG-PLA**
H1	5.70	5.87
H22′	5.91–5.93	6.53
H33′	6.36–6.40	7.62
H44′-H77′-H88′	6.22–6.12–6.15	7.01–7.38
H55′	3.63	4.72
H66′	8.37	-

**Table 5:** Drug encapsulation and release characteristics of CUR loaded in PnBA_30_-*b*-POEGA_70_ and PnBA_27_-*b*-POEGA_73_ micelles in water, without and with ultrasound stimulation.

Sample	DL%	EE%	% Max. Release Rate of CUR without Sonication	% Max. Release Rate of CUR with Sonication
PnBA_30_-*b*-POEGA_70_ + 50%CUR	12	88	9	38
PnBA_27_-*b*-POEGA_73_ + 20%CUR	13	86	15	46

## Data Availability

The data presented in this study are available on request from the corresponding author.

## Supplementary Materials

The following supporting information can be downloaded at: https://www.mdpi.com/article/10.3390/ijms24054621/s1.
