# Supplementary material for: Curcumin-Loaded PnBA-b-POEGA Nanoformulations: A Study of Drug-Polymer Interactions and Release Behavior
Source: Int J Mol Sci. 2023 Feb 27;24(5):4621. doi: 10.3390/ijms24054621 (PMC10003461; doi:10.3390/ijms24054621)
Supplement: Supplementary file 1 [file ijms-24-04621-s001.zip › ijms-2211166-supplementary.pdf]

International Journal of
Molecular Sciences

MDPI

Supplementary Material

Curcumin-loaded PnBA-*b*-POEGA Nanoformulations: A Study of Drug-Polymer Interactions and Release Behavior

Angeliki Chroni [1], Thomas Mavromoustakos [2] and Stergios Pispas [2,*]

1. Results and Discussion

1.1. Physicochemical Characterization of CUR-Loaded PnBA-b-POEGA Nanocarriers

The stability of the CUR-loaded PnBA-b-POEGA solutions was evaluated using DLS measurements. The size, PDI, and mass of the nanocarriers were monitored for a period of one week in aqueous solutions. The results of the DLS stability measurements are provided in Table S1. The data after one week, show that the $PnBA_{30}$-b-$POEGA_{70}$+20% CUR, $PnBA_{30}$-b-$POEGA_{70}$+50% CUR, and $PnBA_{27}$-b-$POEGA_{73}$+20% CUR nanocarriers remained stable, with no significant changes observed in their R_h, PDI, or intensity compared to the measurements on day 1 (Table 2, original manuscript). These findings confirm the stability of the nanostructures and support the efficacy of the formulations.

Table S1. DLS stability results for the CUR-loaded PnBA-*b*-POEGA nanocarriers in aqueous solutions, after one week of storage at 25° C.

Sample	R_h [a] (nm)	PDI [a]	Intensity [a] (a.u)
$PnBA_{30}$-*b*-$POEGA_{70}$ +20% CUR	11	0.25	190
$PnBA_{30}$-*b*-$POEGA_{70}$ +50% CUR	25	0.23	530
$PnBA_{27}$-*b*-$POEGA_{73}$ +20% CUR	25	0.15	880

[a] Determined by DLS after one week at measuring angle 90°.

The analytical techniques of ATR-FTIR and UV-Vis spectroscopy were used to carry out a detailed study of the encapsulation of CUR in the polymeric core of PnBA-b-POEGA micelles. The ATR-FTIR spectra of the $PnBA_{30}$-*b*-$POEGA_{70}$ micelles and CUR-loaded $PnBA_{30}$-*b*-$POEGA_{70}$ nanocarriers are provided in Figure S1a, while those of $PnBA_{27}$-*b*-$POEGA_{73}$ micelles and CUR-loaded $PnBA_{27}$-*b*-$POEGA_{73}$ nanocarriers are displayed in Figure S1b, accordingly. A significant decrease in the ATR-FTIR intensity peaks is observed after 20 wt. % (in the case of the CUR-loaded $PnBA_{27}$-*b*-$POEGA_{73}$ nanocarriers) and 50 wt. % (in the case of the CUR-loaded $PnBA_{30}$-*b*-$POEGA_{70}$ nanocarriers) CUR encapsulation, indicating substantial structural changes in both copolymer/drug systems. Moreover, the ATR-FTIR spectra of the nanocarriers clearly show new peaks specific to CUR (indicated by green annotations. The new characteristic absorption peaks appearing at 1514 cm^{-1} in both Figures S1a and S1b are related to C=C vibrations, originating from the aromatic rings of CUR. Similarly, the absorption peaks at 1590 cm^{-1} are likely caused by C=O stretching vibrations, which are associated with the α,β-unsaturated β-diketone moiety of CUR. In addition, the absorption peaks at 1629 cm^{-1} are correlated with the C=C (conjugated) vibrations of CUR.

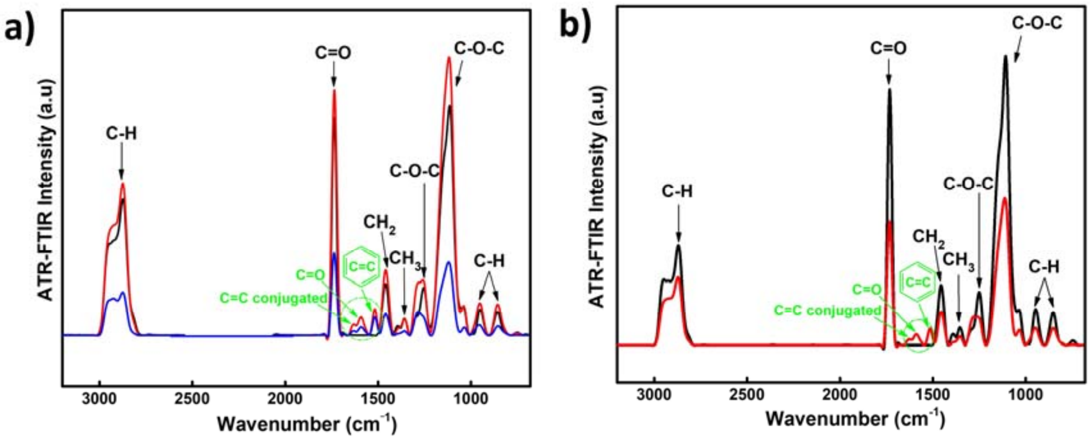

Figure S1. Comparative ATR-FTIR spectra for the a) $PnBA_{30}$-*b*-$POEGA_{70}$ micelles (black line), $PnBA_{30}$-*b*-$POEGA_{70}$ +20% CUR (red line) & $PnBA_{30}$-*b*-$POEGA_{70}$ +50% CUR (blue line) nanocarriers and b) $PnBA_{27}$-*b*-$POEGA_{73}$ micelles (black line) & $PnBA_{27}$-*b*-$POEGA_{73}$ +20% CUR (red line) nanocarriers in aqueous solutions.

UV-Vis measurements provided additional validation of the successful encapsulation of CUR within the PnBA-*b*-POEGA polymeric micelles. The UV-Vis spectra of free CUR in acetone (represented by the black line), $PnBA_{30}$-*b*-$POEGA_{70}$ +20% CUR (represented by the red line) and $PnBA_{27}$-*b*-$POEGA_{73}$ +20% CUR (represented by black line) nanocarriers in aqueous solutions are presented in Figure S2. In agreement with the literature data, the UV spectrum of CUR shows maximum absorption at wavelengths of 260 nm and 420 nm. This is further corroborated by the UV-Vis spectrum of CUR shown in Figure S2 (represented by the black line), which clearly demonstrates the presence of two distinct absorption peaks at 260 nm and 422 nm. Since PnBA-*b*-POEGA diblock copolymers do not exhibit absorption in the relevant UV wavelength range, the presence of two UV absorption peaks (264 nm and 422 nm) in the spectra of $PnBA_{30}$-*b*-$POEGA_{70}$ +20% CUR (red line) and $PnBA_{27}$-*b*-$POEGA_{73}$ +20% CUR (blue line) nanocarriers can be attributed to the UV absorption peaks of the encapsulated CUR. The highest hydrophobicity of the $PnBA_{27}$-*b*-$POEGA_{73}$ micelles, emanating from the increased M_w of the PnBA block (7,800), may underline a greater/stronger CUR encapsulation into the micelles, resulting in an increase in the characteristic UV absorption of CUR at 420 nm compared to the $PnBA_{30}$-*b*-$POEGA_{70}$ +20% CUR nanocarriers.

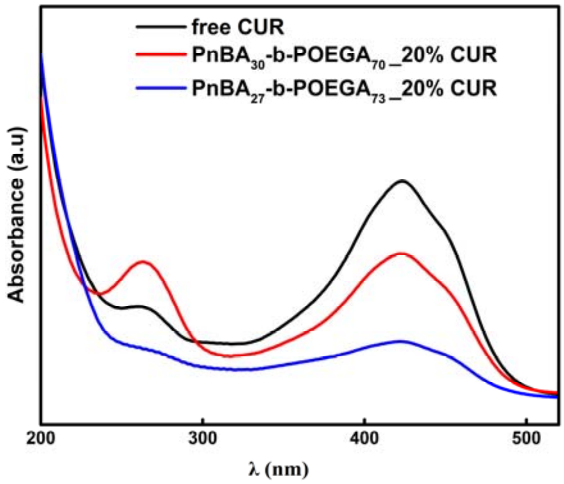

Figure S2. Comparison of UV-Vis spectra for free CUR in acetone (black line), $PnBA_{30}$-*b*-$POEGA_{70}$ +20% CUR (red line) and $PnBA_{27}$-*b*-$POEGA_{73}$ +20% CUR (blue line) nanocarriers in aqueous solutions.

1.2. ^{1}H-NMR Studies on CUR-Loaded PnBA-*b*-POEGA Nanocarriers

The ^{1}H-NMR spectrum of the $PnBA_{27}$-*b*-$POEGA_{73}$ +20% CUR nanocarriers is presented in Figure S3, where the black letters indicate the protons of the copolymer structure, and the red numbers denote the protons of CUR. The distinct proton signal at 5.7 ppm, which is attributed to the vinylic/methine proton of the keto-enol form, clearly indicates the presence of CUR in the presented drug-polymer solutions. In addition, the ^{1}H-NMR spectrum of the nanocarriers, shows the expected proton signals of CUR, confirming its presence in the mixed drug/copolymer solutions and thus implying the successful loading of the drug into the polymeric micelles. The chemical shifts of the proton signals of the $PnBA_{27}$-*b*-$POEGA_{73}$ copolymer in the absence of CUR and in the presence of 20 wt. % CUR (Figure S1) in D_2O solutions are summarized in Table S2.

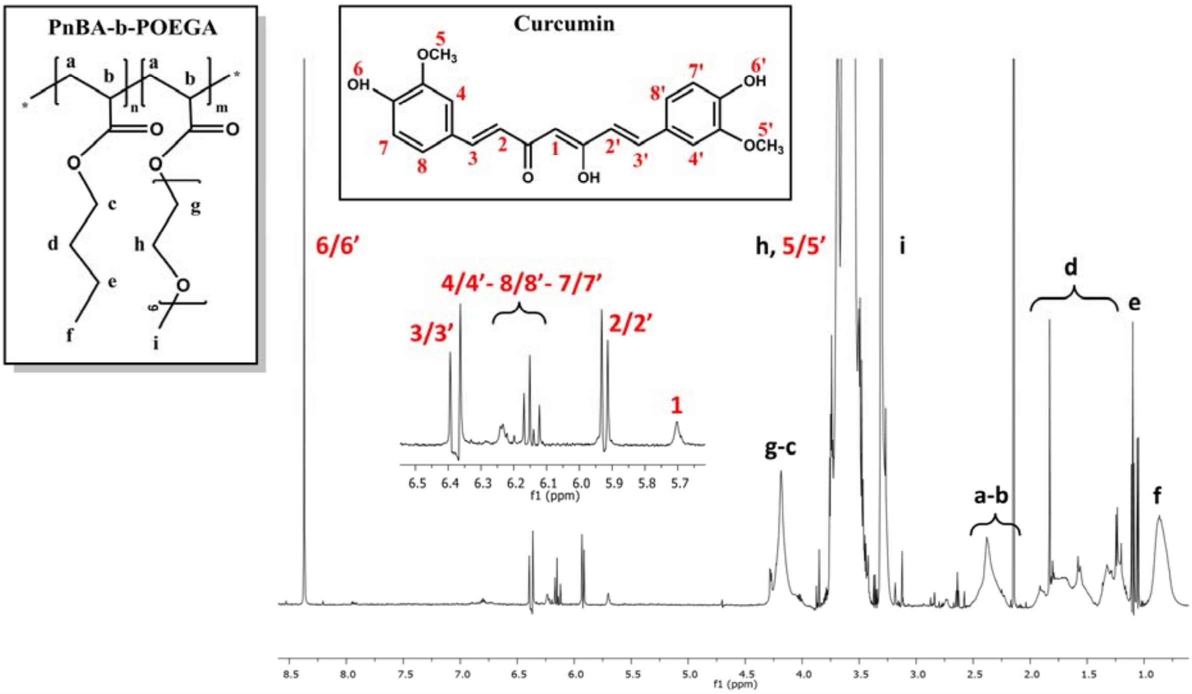

Figure S3. ^{1}H-NMR spectrum for the $PnBA_{27}$-*b*-$POEGA_{73}$ +20% CUR nanocarriers in D_2O solutions, where the black letters denote the protons of the copolymer structure (a–i), the red numbers point to the CUR peaks.

Table S2. ^{1}H-NMR chemical shifts of $PnBA_{27}$-*b*-$POEGA_{73}$ copolymer: a) in the absence of CUR and b) in the presence of 20 wt. % CUR in D_2O solutions.

	**^{1}H-NMR Chemical Shifts of $PnBA_{27}$-*b*-$POEGA_{73}$ (ppm)**	
**Protons of polymer Structure**	**a) in the absence of CUR**	**b) in the presence of 20 wt. % CUR**
Ha-b	2.13	2.14
Hc	3.94	4.05
Hd	1.51	1.52
He	1.1-1.34	1.05-1.33
Hf	0.86	0.85
Hg	4.17	4.17
Hh	3.60	3.62
Hi	3.28	3.29

1.3. 2D-COSY Studies on CUR-Loaded PnBA-b-POEGA Nanocarriers

The 2D-COSY spectrum of the $PnBA_{27}$-*b*-$POEGA_{73}$ +20% CUR nanocarriers is exhibited in Figure S4, where the copolymer protons are denoted by black letters and the CUR peaks are denoted by red numbers. The cross peaks observed between the copolymer and CUR protons provided evidence of intramolecular interactions and facilitated the structural characterization of both the copolymer and CUR.

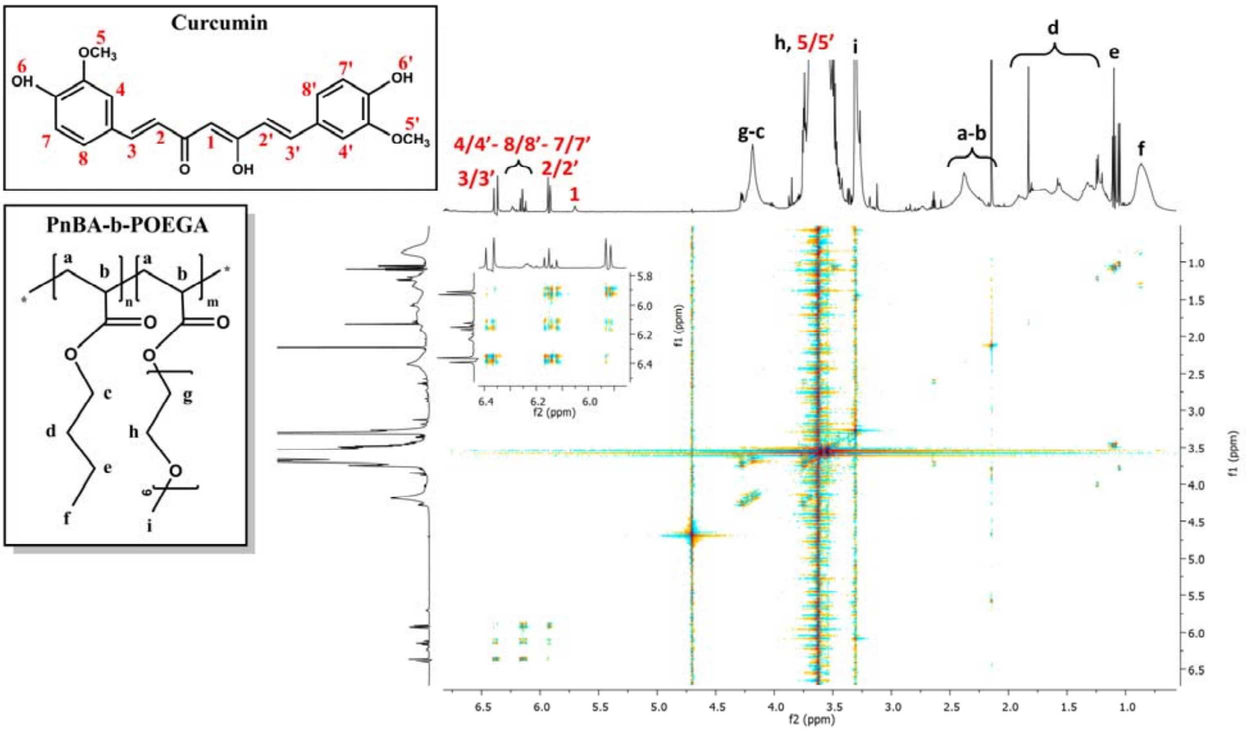

Figure S4. 2D-COSY spectrum of the PnB_{27}-*b*-$POEGA_{73}$ +20% CUR nanocarriers in D_2O solutions, where the black letters (a–i) denote the protons of the copolymer structure, the red numbers point to the CUR peaks.

1.4. 2D-NOESY Studies on CUR-Loaded PnBA-b-POEGA Nanocarriers

The 2D-NOESY results shown in Figure S5 provide information about the binding interactions between CUR and the hydrophobic PnBA core in the $PnBA_{27}$-b-$POEGA_{73}$ +20% CUR nanocarriers. The data suggest that the binding between CUR and the hydrophobic core of PnBA is relatively weak, as only the aliphatic linker of CUR (2/2') is in close proximity to the butyl chain of PnBA at position e. This implies that hydrophobic interactions between the aliphatic carbon linker of CUR and the butyl chain of PnBA are responsible for the binding.

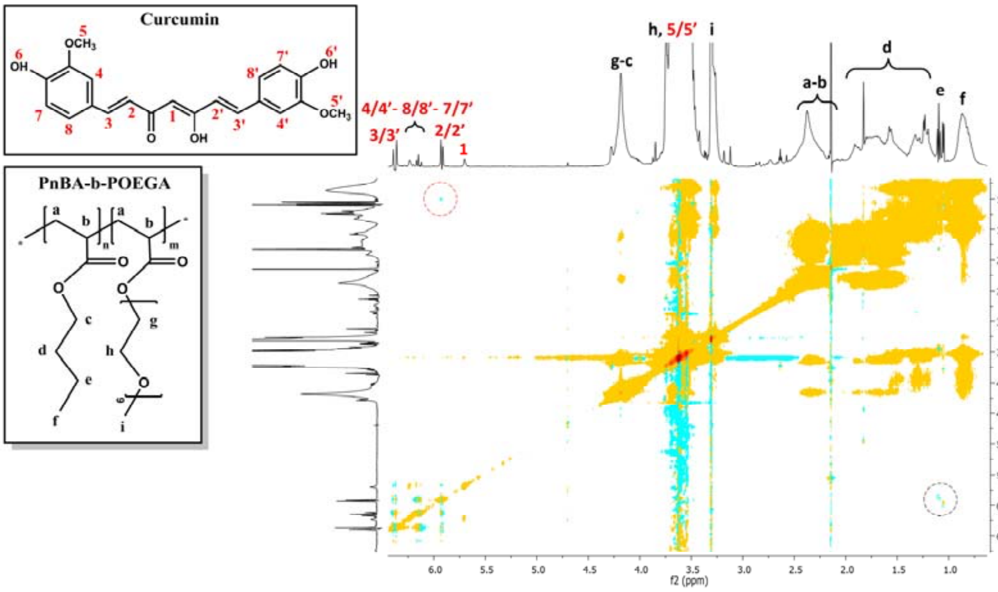

Figure S5. 2D-NOESY spectrum of the $PnBA_{27}$-*b*-$POEGA_{73}$ +20% CUR nanocarriers. The black letters (a–i) denote the protons of the copolymer structure, the red numbers point to the CUR peaks.

1.5. 2D-DOSY Studies on CUR-Loaded PnBA-b-POEGA Nanocarriers

Figure S6 shows the diffusion experiments for the $PnBA_{27}$-b-$POEGA_{73}$ +20% CUR nanocarriers. Three sets of traces with medium constants ($D = 4.9 \times 10^{-7}$ m^2 s^{-1} and $D = 9.5 \times 10^{-6}$ m^2 s^{-1}) appear in the 2D-DOSY spectrum of the nanocarriers, which can be attributed to the diffusion of block copolymer micelles and unimolecular block copolymers, respectively. The trace with the highest coefficient ($D = 1.02 \times 10^{-5}$ m^2 s^{-1}) can be related to the diffusion of CUR. The 2D-DOSY results of the $PnBA_{27}$-b-$POEGA_{73}$+20% CUR nanocarriers (Figure S6) demonstrate a pronounced exchange rate between the free and micelle-bound state of CUR. This is evidenced by the observed diffusion coefficients, which suggest a dynamic and fluid equilibrium between the two states.

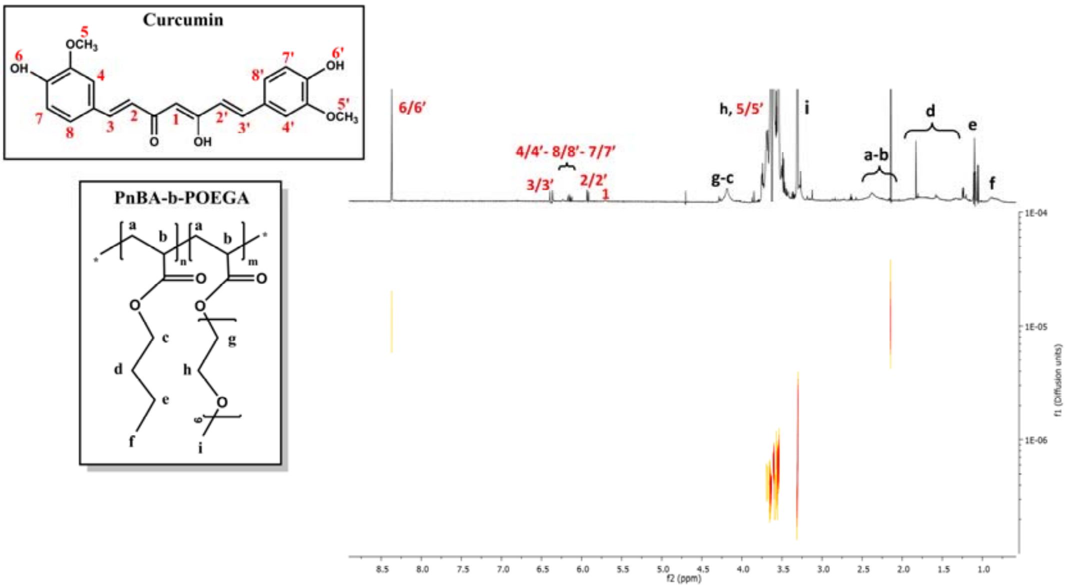

Figure S6. 2D-DOSY spectrum of $PnBA_{27}$-*b*-$POEGA_{73}$ +20% CUR nanocarriers. The black letters (a–i) denote the protons of the copolymer structure, the red numbers point to the CUR peaks.
